# Structural representations: causally relevant and different from detectors

**DOI:** 10.1007/s10539-017-9562-6

**Published:** 2017-02-02

**Authors:** Paweł Gładziejewski, Marcin Miłkowski

**Affiliations:** Institute of Philosophy and Sociology, Polish Academy of Sciences, Nowy Świat 72, 00-330 Warsaw, Poland

**Keywords:** S-representation, Mental models, Mental representation, Antirepresentationalism, Interventionism about causation, Mechanistic explanation

## Abstract

This paper centers around the notion that internal, mental representations are grounded in structural similarity, i.e., that they are so-called S-representations. We show how S-representations may be causally relevant and argue that they are distinct from mere detectors. First, using the neomechanist theory of explanation and the interventionist account of causal relevance, we provide a precise interpretation of the claim that in S-representations, structural similarity serves as a “fuel of success”, i.e., a relation that is exploitable for the representation using system. Then, we discuss crucial differences between S-representations and indicators or detectors, showing that—contrary to claims made in the literature—there is an important theoretical distinction to be drawn between the two.

## Introduction

Antirepresentationalism has been one of the major recent trends in theorizing about the mind. Some modern antirepresentationalists employ a sort of trivializing argumentative strategy. That is, instead of (or in addition to) developing new theories of cognition that do without the notion of representation, they attempt to show that some of the most prevalent existing notions of mental representation are not suited to do the theoretical jobs that are expected of them. In particular, the idea that representations are covariance-based indicators or detectors has been subjected to this sort of trivializing attack. It has been argued that detectors functionally boil down to mere causal mediators, and thus are not representations (Ramsey 2007), or that they are not contentful (Hutto and Myin 2013).

One way that representationalists might want to oppose attempts to trivialize representations is to defend the *status quo*, for example, to argue that detectors are representations after all. But perhaps a different reaction is more justified and fruitful. Perhaps representationalists should treat the “trivializing” strand of antirepresentationalism as an opportunity to develop, strengthen, and indeed reform the mainstream understanding of what representations are so that the resulting new notion is no longer subject to the trivializing arguments. In fact, something like this sort of reaction is starting to take shape in the literature. This is because in parallel to antirepresentationalism, another trend in theorizing about representation has recently gained momentum, wherein people move away from seeing mental representations as indicators or detectors, and towards construing them in terms of internal models (Bartels 2006; Grush 2004; Gładziejewski 2016a, b; Isaac 2012; O’Brien 2014; O’Brien and Opie 2004; Ramsey 2007; Rescorla 2009; Ryder 2004; Shagrir 2012; Shea 2014; for earlier treatments, see also Craik 1943; Grush 1996; Cummins 1996). Here, the model is understood as an internal structural representation, i.e., a representation grounded in structural similarity^1^ between the representation itself and its target. Here, we want to address two crucial issues that arise in the context of this approach to representation.

Our first concern is with the idea that S-representations come into play when structural similarity can be actively exploited or relied upon by a cognitive system or mechanism. In other words, similarity relations that give rise to S-representations are *exploitable* similarities (Godfrey-Smith 1996; Shea 2007, 2014). There are at least two reasons to think that the notion of exploitable similarity is of crucial importance for theorizing about S-representation. First, it demonstrates that S-representations do not have to be construed in terms of passive, pragmatically detached “mirrors of nature.” Second, the notion can prove useful in resolving problems that seemingly plague similarity-based theories of representation. For example, one might claim that because of the ubiquity and “cheapness” of structural similarity, similarity-based theories imply a problematic panrepresentationalism. But this can be avoided if we cut down the class of representation-relevant similarities to *exploitable* similarities (see Ramsey 2007; Shea 2007, 2014). However, even though the very notion of similarity as an exploitable relation is by no means new in the literature, to our knowledge it has never been elaborated in detail. Some authors (e.g., Ramsey 2007) characterize the role of similarity by appealing to explanatory considerations, i.e., to the fact that invoking similarity is sometimes necessary to understand the successful operations of cognitive agents. But as it stands, this approach seems compatible with views which treat representation talk as a purely instrumental “gloss” (Egan 2014), or ones that treat representational claims as fictions, i.e., as not literally true (see Sprevak 2013). In the present paper (“Exploiting structural similarity: a mechanistic-interventionist account” section), we want to propose a precise, as well as more metaphysically committed interpretation of exploitable similarity, one which results in a firmly realist treatment of S-representations. Using the neomechanist theory of explanation and the interventionist theory of causal relevance, we claim that exploitable similarity is *causally relevant* for the successful operation of a cognitive mechanism (“Exploitable similarity as causally relevant similarity” section). Then we show that this causal role is indeed properly attributed to the relation of similarity as such, and does not boil down to the role played by the representational vehicle alone (“Is *similarity* really causally relevant?” section).

The second aim of the paper is to critically examine the recent skepticism about whether S-representations are in fact distinct from detectors or indicators. In particular, Morgan (2014) has argued that under scrutiny, S-representations turn out to be functionally equivalent to detectors. If this is true, it would mean that there is no theoretically significant distinction to be made between the two. In particular, it would turn out that it is a mistake to think that S-representations act as internal models, which, on the face of it, is importantly different from acting as an indicator or detector. Furthermore, if S-representations are in fact indistinguishable from detectors, then it could be argued that the former fall under the same trivializing arguments as the latter. Nonetheless, we will argue (in “S-representations versus. detectors revisited” section) that there is an important distinction to be made here after all. We start by presenting the reasoning behind the criticism of the S-representation/detector distinction, and then lay out a way of delineating S-representations from detectors. Our proposal appeals to (1) the crucial role that the similarity of structures plays in S-representations but not in detectors, and (2) the fact that S-representing, as a strategy of guiding action and cognition, is not purely reactive (as is the case with mere detectors), but involves an endogenous source of control.

## Exploiting structural similarity: a mechanistic-interventionist account

### Exploitable similarity as causally relevant similarity

It is usually recognized that the mere existence of structural similarity between two entities is by no means sufficient to confer on one of those entities the status of representation. S-*representations* only come into play when a cognitive system depends, in some nontrivial sense, on the relation of similarity in its engagements with its representational targets. As Godfrey-Smith (1996) and Shea (2007, 2014) put this, the correspondence (here, the structural similarity) between representation and its target should be understood as “fuel for success” or a resource that enables organisms to “get things done” in the world. In other words, similarity should be understood as a relation that is *exploitable* for some larger representation-using system. We now want to address the question of what it means exactly for structural similarity to be exploitable. In particular, we will try to clarify this idea in the context of purely subpersonal S-representations of the sort that we could find inside a mechanical system such as a human brain.

Let us start by taking a closer look at the basic, commonsense intuition that underlies the notion of exploitable similarity. Consider an external, artifactual S-representation such as a cartographic map. We can at least sometimes explain someone’s success at navigating a particular territory by pointing to the fact that the person in question used an accurate map of this territory (and vice versa, we can explain someone’s navigational failure by citing the fact that the person in question used an inaccurate map). Users of cartographic maps owe their success to the similarity that holds between the spatial structure of the representation and the spatial structure of the territory it represents (analogously, the failures can be due to the lack of similarity between the representation and what is represented). This link between similarity and success generalizes to all S-representations, including, we claim, the ones that do not require interpretation by a human being.

On the view we are proposing, explanations of success that invoke the similarity between the representation and its target can be true in virtue of similarity being *causally relevant* to success. That is, the structural correspondence can quite literally cause the representation-user to be successful at whatever she (or it) is using the representation for, and lack of structural correspondence can cause the user to fail at whatever she (or it) is using the representation for. Explanations that invoke S-representations should thus be construed as causal explanations that feature facts regarding similarity as an explanans and success or failure as an explanandum. To exploit structural similarity in this sense is to use a strategy whose success is causally dependent on structural similarity between the representational vehicle^2^ and what is represented.

Our treatment invokes two concepts that are in need of clarification, especially when applied to internal, subpersonal representations: the notion of success/failure (for which similarity is causally responsible), and the notion of causal relevance. We will now concentrate on each of these notions in turn. Let us start with success and failure.

The idea that *human agents* can succeed or fail at whatever they use S-representations for seems straightforward enough and we will not dwell on it here. But how to understand success/failure in the case of internal, subpersonal representations of the sort that are of interest to us here? We propose to look at the problem through the lens of the prominent neomechanistic theory of explanation, as applied to cognitive-scientific explanation (Boone and Piccinini 2015; Bechtel 2008; Craver 2007; Miłkowski 2013). Neomechanists see the cognitive system as a collection of mechanisms. A mechanism is a set of organized components and component operations which jointly enable the larger system to exhibit a certain phenomenon (often understood as a capacity of this system). Mechanisms in this sense are at least partly individuated functionally, that is, by reference to the phenomenon that they give rise to—they are essentially mechanisms *of* this or that cognitive function (mindreading, motor control, attention, perceptual categorization, spatial navigation, etc.). Components and operations derive their functional characterization from the function of the larger mechanism they are embedded in. That is, the function of a component is determined by an operation such that it is through the performance of this particular operation that the component in question contributes to a phenomenon for which the larger mechanism is responsible (see Craver 2007). This is why, say, the function of the heart as a component of a mechanism responsible for blood circulation lies in its pumping blood, and not in its emitting rhythmic sounds; it is the former, and not the latter operation through which the heart contributes to blood circulation.

The vehicles of internal S-representations can be treated as components of cognitive mechanisms, and are targets of various cognitive operations. Each mechanism equipped with an S-representation as its component part underlies a certain phenomenon, i.e., some cognitive capacity. S-representations construed as mechanism components owe their functional characterization to how they contribute to the phenomenon that the larger mechanism is responsible for. What we mean by this is, essentially, that *structural similarity* between the representation and what it represents is what contributes toward the mechanism’s proper functioning. To put it more precisely, any mechanism responsible for some capacity C which includes an S-representation as its component can *fail* to realize or enable C as a result of the fact that the component in question is not (sufficiently) structurally similar to the representational target; and analogously, when the mechanism *succeeds* at realizing or enabling C, this is at least in part due to the fact that this component is (sufficiently) structurally similar to the target. So structural similarity is causally relevant to success/failure because the ability of any S-representation-involving mechanism to perform its function depends on the degree of structural similarity between the representational vehicle and the target. Success and failure are treated here as success or failure at contributing to some *function or capacity of a mechanism*.

We now turn to the question of what it means for similarity to be causally relevant to success (or failure) thus understood. Here we aim to make use of James Woodward’s (2003, 2008) popular interventionist theory of causal relevance.^3^ It is beyond the scope of the present discussion to present Woodward’s theory in detail so a rough sketch will have to suffice. The core idea behind the interventionist view is that claims of causal relevance connect two variables, say, X and Y.^4^ What it takes for X to be causally relevant to Y is that appropriate interventions into X (i.e., interventions that change the value of X) are associated with changes in Y (i.e., the values of Y):

**(M)** X causes Y if and only if there are background circumstances B such that if some (single) intervention that changes the value of X (and no other variable) were to occur in B, then Y would change. (see Woodward 2003, 2008)

The intervention in question can be helpfully understood as an experimental manipulation of X in controlled settings, although Woodward’s theory does not require human agency to be involved in establishing causal relations—any change of the value of X could potentially count as an intervention, even one that is not dependent at all on human action. Importantly, there are certain conditions that an intervention must meet in order to establish a causal connection between X and Y. For example, the intervention must not change the value of Y through any causal route except the one that leads through X (e.g., it must not change the value of Y directly or by directly changing the value of a variable that mediates causally between X and Y) and it must not be correlated with any causes of Y other than X or those that lie on the causal route from X to Y.

By employing the interventionist view, we can now understand the causal relevance of similarity for success in the following way. The structural similarity between the representational vehicle and the target is causally relevant for success by virtue of the fact that interventions in similarity would be associated with changes in the success of whatever capacity that is based on, or guided by the representation in question. That is, manipulations on similarity would also be manipulations on the ability of the representation-user—be it a human being or some internal cognitive mechanism—to be successful at whatever she or it is employing the representation for.

To make this proposal more precise, let us apply (M) to the similarity-success relation. The variable X corresponds to similarity between the vehicle and what is represented. It would probably be a gross simplification if we treated X as a binary variable, with one value corresponding to the existence, and the other to the lack of similarity. Luckily, structural similarity can be easily construed as a gradable relation, depending on the degree to which the structure of one relatum actually preserves the structure of the another relatum (see note 1; for another account that explicitly defines similarity as coming in degrees, see: Tversky 1977; Weisberg 2013). This way we can treat X as capable of taking a range of values {X_1_, X_2_,…, X_n_}, where each increasing value corresponds to an increased degree of similarity between the vehicle and the target. Therefore, between the lack of any similarity and a complete structural indistinguishability, there is a range of intermediate possibilities.

What about Y, the variable that corresponds to success/failure? As far as we can see, S-representations could turn out to feature in a diverse set of mechanisms which give rise to a diverse set of cognitive functions, like motor control and motor planning, perceptual categorization, mindreading, decision making, etc. Now, cognitive systems can be more or less *effective* at realizing each such function: they can perform better or worse at motor control and planning, perceptually categorizing objects, attributing mental states, making decisions, etc. In this sense, we can treat the variable Y as corresponding to degrees of success of the mechanism in question at enabling an effective performance of a given capacity. Increasing values of Y = {Y_1_, Y_2_,…, Y_n_} would correspond to increasing degrees of success thus understood. But what sorts of values can we have in mind exactly? Here we want to remain as open as possible. Any scientifically respectable way of measuring success can do. For example, the success could be measured by the average frequency of instances of a certain level of performance at some cognitive task, or the probability of a certain level of performance at some task, or a distribution of probabilities of possible levels of performance at some task, etc. The details will always depend on the sort of function in question, as well as on the experimental paradigm used to test or measure it.

We may now formulate our thesis as follows. For similarity to cause success, interventions into the value of X (which corresponds to the degree of structural similarity between the representational vehicle and what it represents) should result in systematic changes in the value of Y (which corresponds to the degree of success of the mechanism that makes use of an S-representation in performing its mechanistic function or capacity). In particular, by intervening in X so that its value increases, we should increase the value of Y; and by intervening in X so that its value decreases, we should decrease the value of Y.

Before we move on, it needs to be noted that the relationship between similarity and success is nuanced in the following way. Good S-representations resemble relevant parts of the world only partially. Maps never mirror the territory in all its detail; instead, they are intentionally simplified, selective, and even distorted. The same applies to subpersonal S-representations. There are at least two reasons to think that. First, S-representations that resemble the target too much become excessively complex themselves. We should then expect there to be a trade-off between a representation’s structural complexity and the temporal or computational resources (costs) that real-life cognitive systems have at their disposal. It is doubtful that limited agents could generate S-representations that come even close to mirroring the structural complexities of the world. Second, in a world as complex as ours, generating maximally accurate S-representations tends to result in overfitting the data, which decreases the representation’s predictive value (this latter point applies to S-representations that are statistical models of the environment).

This general observation can be expressed using our preferred interventionist framework. Suppose that increasing values of variable X correspond to increasing structural similarity between the vehicle and what is represented, and the increasing values of variable Y correspond to increasing success. Now, to accommodate our point, we may say that although in real-life cases of S-representation, there is a positive causal relation between X and Y, it only holds within a limited range of values of X. For simplicity, we may suppose that the relation holds from the lowest value of X to some specific larger value, but it disappears when X exceeds this value. That is, once the value of X exceeds a certain level, then (e.g. due to low cost-effectiveness or overfitting) its relationship to Y breaks down, e.g. increasing the value of X may begin to decrease the value of Y. Crucially, the lesson to be drawn here is not that similarity is functionally irrelevant, but simply that too much similarity can render the S-representation inefficient at serving its purpose. Our proposal is therefore that structural similarity is causally relevant only in a certain range, and the exact range depends on the overall structural trade-offs of the similarity-based system.

The following empirical illustration should illuminate our view. In the philosophical literature, hippocampal spatial maps in rats have been proposed as a good example of an internal S-representation (Ramsey 2016; Rescorla 2009; Shea 2014). The rat’s hippocampus is thought to implement an internal map of the spatial layout of the environment, encoded in a Cartesian coordinate system. According to this hypothesis, the co-activation patterns of so-called place cells in the hippocampus correspond to the spatial structure of the rat’s environment (Shea 2014). That is, the pattern of co-activation relationships between place cells (roughly, the tendency of particular cells to show joint activity) resembles the structure of metric relations between locations within the environment.^5^ This hippocampal map constitutes a component of a cognitive mechanism which underlies the ability to navigate the environment (Craver 2007). The rat’s capacity to find its way within the environment, even in the absence of external cues or landmarks, depends on the fact that it has an internal mechanism equipped with a map of the terrain. This capacity for navigation is usually tested by verifying the rat’s ability to find a reward (food) within a maze in which the animal has no reliable access to external orientation points (see Craver 2007; Redish 1999 for reviews).

As has been already argued in the literature, spatial navigation using hippocampal maps is an instance in which the structural similarity between the map and the territory is being actively exploited by the organism (Shea 2014). Similarity serves as a resource that the rat depends on in its dealings with problems that require spatial navigation. Our proposal provides what we think is a clear and precise interpretation of this claim. The map-world similarity is causally relevant to the rat’s success at finding its way in the environment. This means that we could manipulate the rat’s capacity to navigate in space by intervening in the degree to which its internal map resembles structurally (the relevant part of) the environment. We know, for example, that rats are quite efficient at constructing and storing separate maps for particular mazes (Alme et al. 2014). We may imagine an experiment in which we place the rat in a previously-learned maze and then intervene on the co-activation structure of place cells in a way that distorts (i.e., decreases) the structural correspondence between the map and the maze to a particular degree. If the similarity is really being exploited, then intervention of this sort should decrease the rat’s ability to navigate the particular territory, and we should be able to observe and measure this decrease by investigating the change in the rat’s performance at finding rewards in the maze. What is more, the rat’s navigational capacity (variable Y) should be reduced to a degree which is in proportion to the degree to which we decreased similarity (X) between its internal map and the spatial structure of the maze. And crucially, our intervention should change the rat’s performance *only insofar as it constitutes an intervention on similarity as such*.

### Is *similarity* really causally relevant?

The following issue might well be raised in the context of our mechanistic-interventionist treatment of the notion of exploitable similarity. One could wonder whether it is really similarity *as such* that is causally relevant to success. Notice that it is impossible to perform an intervention on the similarity relation in any way other than by intervening in the structure of at least one of its relata (here, the representational vehicle or the represented target). But this invites a worry. Would it not be much more parsimonious to simply state that what is causally relevant for success are structural properties of the vehicle and/or the target? After all, it is by intervening in either of them that we manipulate success. Why bother attributing the causal role to *similarity* itself? For example, to change a rat’s performance at navigating mazes, it will suffice to intervene on the hippocampal map. Why not simply say that it is the structure of the map (the representational vehicle) that is causally relevant to the rat’s success at spatial navigation? Why treat the *relation* between the map and the environment as causally relevant?

To reply to this objection, we need to be careful to make the distinction between interventions that change the way some cognitive system *acts* (behaviorally or cognitively) and interventions that change the *success* of its actions. The change of action can, but does not have to change the success of the organism at whatever it is doing. If the change in the way the system acts is accompanied by an appropriate change in the external environment, the success can stay at the same level (e.g., we could change the rat’s behavior in a maze without changing its ability to find food if the maze itself changes accordingly). At the same time, the same manipulation of action can change the success of the organism either by increasing it or decreasing it—again, the direction of influence will depend on properties of the environment (e.g., on the structure of the maze that the rat is traversing). So there is no context-free, one-to-one correspondence between action and success. The reason for this is that success and failure in the sense we are using are essentially *ecological* categories. They co-depend both on what a given system is doing, *and* on the world within which it is doing it.

Notice now that by concentrating solely on the properties of the representational vehicle, we would completely miss the point just made. Surely, interventions in the structural properties of the vehicle (e.g., the hippocampal map) would change the cognitive system’s *actions* (e.g., the rat’s behavior when placed in a maze). That much is not debatable. But manipulating actions is not the same as manipulating success. Because of this, the effect that the structure of the vehicle has on action does not imply that the same sort of relationship exists between the vehicle’s structure and *success*. It is impossible to say how manipulating the vehicle’s structure (and so the organism’s action) will change success independently of facts about the target; or more precisely, independently of the facts regarding structural similarity between the vehicle and the target. In other words, interventions on the vehicle’s structure change the success *only insofar as they change the degree of similarity between the vehicle and the target*. They increase success if they increase the structural fit between the vehicle and the target. They decrease success only if they decrease the structural fit. And they do not change the success if they do not bring about any change in the structural fit. In any case, what the success depends on is not just the vehicle, but also structural similarity. Of course, again, the only way to intervene on similarity is by manipulating the relata. But it is just wrong to conclude from this that similarity itself is not what is causally relevant here.

Let us formulate our point using some technicalities of Woodward’s account of causal relevance. Suppose that the independent variable X corresponds *not* to similarity between the vehicle and the target, but to purely vehicular-structural properties of the representation. More precisely, imagine that each value of X corresponds to a different potential structural pattern of the vehicle, regardless of its relationship to anything outside the mechanism. The dependent variable Y remains the same, i.e., it measures the degree of success at realizing some capacity. Now, there are certain constraints that Woodward (2003) puts on any scientifically respectable causal relationships. Two of them are relevant for our present purposes. First, interventions should not simply effect *some* changes in Y. Rather, the relation between X and Y should be systematic in that we should be able to establish which values of X correspond to which values of Y. Second, the relationship between X and Y should be stable, viz. it should hold across a wide range of different background conditions. But notice that neither of those constraints is met on the interpretation of X and Y that we are now considering. First, because of the reasons we mentioned above, there is no clear mapping from values of X to values of Y, which prevents the relationship between those variables from being systematic in the relevant sense. Setting X at some value could well increase the value of Y, decrease it or even not change it all. Second, the relation between X and Y is by no means stable. In fact, it is fundamentally unstable because of how dependent it is on the states of the environment. It is not possible to say how manipulation of X will change the value of Y independently of the state of the target. Again, the same manipulation of X (e.g., setting the structure of the spatial map in the hippocampus) could bring about drastically different results depending on external circumstances (e.g., depending on the spatial structure of the maze that the rat navigates).

Both Woodward’s constraints are however met if we go back to our original view and consider the variable X to correspond to the degree of *similarity* between the representational vehicle and the target. The relation between X and Y is then both systematic and stable. It is systematic because we can map increasing values of X onto increasing values of Y. And it is stable at least in the sense that it cannot be broken down by changes in the target. After all, the value of X itself partially depends precisely on properties of the target.^6^ Overall, we think that these considerations provide strong reasons to think that in an S-representational mechanism, what is causally relevant to success is really the relation of structural similarity.

## S-representations versus detectors revisited

Let us now turn our attention to the problem of distinguishing S-representations proper from mere detectors or indicators. Some authors challenge the very notion that there is a genuine distinction to be made here (Morgan 2014) because they think that one cannot differentiate systems or mechanisms that operate on the basis of covariance from ones that exploit structural similarity. It is claimed that when some system or mechanism operates by using a detector whose states reliably covary with states of the target, it is straightforwardly possible to show that the system or mechanism in question relies on the similarity that holds between the detector’s structure and some target. Consider the notorious thermostat, equipped with a bi-metallic strip whose shape reliably reacts to (hence, covaries with) variations in the ambient temperature, and, in turn, switches the thermostat’s furnace to keep the temperature at a certain level. It is usually claimed that *if* it is even justified to treat it as a representation (which is far from uncontroversial in itself, see (Ramsey 2007, Ch. 4), the bi-metallic strip counts as, at most, a detector or an indicator of some state of affairs. However, on closer inspection, it turns out that reliable causal covariance is not the only relation that connects the strip to ambient temperature. They are also related by way of structural similarity (see also O’Brien 2014). Namely, there exists a structure-preserving mapping between the pattern of the bi-metallic strip’s possible shapes and the pattern of possible variations in ambient temperature. Furthermore, it may seem that the thermostat would not have the ability to adapt its behavior to the changing environment without the existence of a mapping between the metal strip and ambient temperature. Perhaps, then, we should regard the thermostat as a device that makes use of an S-representation after all? Or is there a genuine theoretical distinction to be made here at all? Maybe the conclusion to make is that detectors “just are” S-representations (Morgan 2014)?

This is a serious challenge for anyone who wants to see construing representation in terms of exploitable similarity as significantly different, and perhaps also deeper in some important ways than construing it in terms of indication or detection. Nonetheless, we want to argue that there is, in fact, a principled way of drawing the distinction between S-representations and detectors. We claim that there are two fundamental differences between S-representations and detectors in virtue of which the distinction is justified. The first difference pertains to the fact that that although detectors can exhibit structural resemblance to their targets, this relation is not relevant for their workings in quite the same way that it is relevant in the case of S-representations proper.^7^ The second difference relates to what distinguishes the *functioning* of S-representations from the way detectors function. We discuss those differences in turn.

Regarding the first difference, let us reconsider the thermostat example. One can ascribe the structure to the bi-metallic strip because of the relations between different shapes that it can take depending on the surrounding temperature. But notice that for a system such as the thermostat, it is not *necessary* or *essential* for the relational structure of possible indicator states to replicate the relations between different variants of ambient temperature in any particular way. Of course, it *is* the case that, say, the strip curvature that indicates 33 °C is closer to the one that indicates 34° than to the one that indicates 17°. But we can imagine an intervention in a thermostat which breaks this structural resemblance while leaving the thermostat’s workings intact. We may imagine that following this intervention, the detector strip reacts to the temperature being 33° by taking shape that is closer to the one that corresponds to 17° than to the one that corresponds to 34°. However, this fact is simply irrelevant as long as the 33-degree-detector-state is specific to the environmental circumstances, such that (1) the detector enters this state as result of the temperature being 33°, (2) it switches the furnace into a state that is appropriate or functional given the temperature being 33°. The *relation* that this state bears to other states is irrelevant or accidental. To generalize, in detectors or indicators, the relations between alternative detector states need not replicate the relational pattern of the target. In this sense, the relation of structural similarity is epiphenomenal in their case: as such, it does not play a role in enabling the detector system to work properly.

By contrast, an S-representation cannot do its job (i.e., enable success) without being structurally similar to the target. Here, the pattern of relations between components of the S-representation plays a crucial role. For example, a map—be it artifactual map or neurally-realized cognitive map—needs to stand in a structural resemblance relation to the terrain if it is to perform its S-representational job; and any figure placed within a map can act as an S-representational surrogate only insofar as it stands in certain relations to other figures or lines on the map. In other words, in S-representations, the *structure* (i.e., the relational pattern) of the vehicle—and the resemblance that it bears to the target structure—plays a major role which is missing in the case of detectors.

The second difference that underlies the S-representation/detector distinction does not pertain to the nature of the relation that connects the representational vehicle to its target. Rather, it relates to what distinguishes the *functioning* of S-representations from the way detectors function.

Let us start with a simple illustration. Consider two people facing the problem of navigating their way from one location to another in a city. Person A has traversed this route many times in the past, to the point that she does not need to elaborately plan how to reach her destination. All that she has to do is to react, at appropriate times and in appropriate ways, to particular environmental cues and landmarks (say, by turning left upon seeing a church, then right at the second crossing, etc.). Her navigational choices are fully dictated or determined by the territory itself: all that she does is respond to it in ways which enable her to eventually reach the destination. Now, person B has no previous experience with the city and so traverses the same route using satellite navigation. In the case of person B, it is not possible to explain her success at reaching the destination by simply pointing to how she *reacts* to environmental cues. This is because her ongoing navigational decisions depend on what happens in the navigation system. What guides B’s actions are the system-derived anticipations and instructions, not the world itself. There surely are purely “receptive” aspects to using satellite navigation—B obviously needs to interact with the environment itself in order to verify satellite-based suggestions, and the system’s receiver must itself interact with the satellite to track B’s current position. However, there is an important sense in which person B’s actions are controlled by the satellite navigation itself, as opposed to being fully controlled by the terrain.

Here is our point. The strategy employed by person A is representative of what is crucial, from a functional standpoint, to cognitive strategies that employ detectors. In detector-based strategies, the represented part of the world constitutes the locus of control of an action or a cognitive process. Detectors are functionally bound to their targets. This is because all the there is to working as a detector or indicator is to be *causally selective* in useful ways. Detectors tend to react exclusively to certain states of affairs, and, in turn, generate cognitive or behavioral responses that are appropriate given the circumstances. This the case with the thermostat as a detector-based mechanism: its bi-metallic strip reacts or responds to the target in a way that is useful to the larger mechanism. The way the thermostat behaves is under the control of the environment itself.

One important thing to note here is that the “causal selectivity” at issue can be realized in ways other than through direct causal relation between the detector and its purported target. It could be established by indirect, mediated causal chain, or by the detector and the target sharing a common cause. In yet other cases, the detector may be causally related to a state which is merely spatiotemporally correlated with the target. Take, for instance, magnetosomes in magnetotactic bacteria. Magnetosomes react causally to the magnetic North; but given that the magnetic North is reliably correlated with the location of oxygen-free water, they can drive the bacteria towards its preferred environment. Here the detector is already causally disconnected from what it supposedly detects (the target). Still, its workings boil down to being reactive to the environment in useful ways.

On the contrary, using S-representations is not a matter of simply selectively reacting to targets. In our toy example, this is apparent in person B’s case in that it is not the environment itself, but rather what happens in satellite navigation that constitutes the locus of control of the navigation process. To generalize this point, what is characteristic of S-representation-based strategies is that they employ an internal or endogenous source of control over action or cognitive processes; they are, so to speak, active, and not simply reactive strategies (for an illuminating discussion of endogenously active mechanisms in nature, see Bechtel 2008, 2013). Furthermore, what is also crucial is that processes or manipulations over S-representations exhibit a certain degree of functional freedom from their targets—freedom which is absent in the case of detectors. That is, the way the S-representation gets manipulated or updated is endogenously controlled; it depends on the internal set-up of the S-representations itself and is not dictated (although it may be affected) by the causal coupling with the target. Again, what satellite navigation system anticipates to be the case as one traverses the terrain is not (just) a matter of what happens in the world, but (also) of what is encoded in the map itself.

One of the major consequences of S-representations’ being endogenously-controlled and functionally free in the sense described above is that they can naturally perform their duties even when the processes that they undergo do not correlate with any concurrent target or represented processes; that is, when the S-representation cannot be said to *track* anything that actually takes place. In other words, S-representations can perform their function even if they do not change “in response” to targets—at least on any useful or illuminating interpretation of what “in response” could possibly mean in this context. Take S-representations which function in a robustly off-line manner. In such cases, the represented entity could be so spatiotemporally distant from the representation user so as to count as absent for it (Clark and Toribio 1994). Just think of a person who manipulates an interactive digital map in order to plan a *future* trip, or even to consider some route purely *counterfactually*. In this strong sense, deploying S-representations off-line consists of manipulating them for the purpose of representing things located in the distant past, future, or ones that are merely counterfactual. Notice also how in the case of (S-)representing future and counterfactual states of affairs, there is no possibility—at least not without some serious metaphysical gymnastics—of saying that the representation is reactive to what is represented (after all, the latter is nonexistent at the time the representation is employed).^8^

One might raise the question of how this functional story about S-representations relates to the story about exploitable similarity. To answer this issue, we need to note that being an endogenously-controlled process is not *sufficient* for this process to count as employing S-representation. Exploitable similarity needs to be involved.

Let us elucidate this point by showing how structural similarity is exploited in cases of S-representations that are used off-line, this time concentrating on subpersonal representations of the sort that could feature in cognitive mechanisms. Three ingredients are involved in the off-line use of an S-representation thus construed. First, the S-representation is actively transformed or manipulated within the mechanism. That is, the S-representational vehicle undergoes an endogenously-controlled process in which its structure changes over time. The structure of the vehicle is being effectively put to use. Second, manipulations of this sort are employed by the larger mechanism to perform a certain function. For example, the effects of manipulations could serve (for some consumer component) as a basis for a decision about which course of action—out of some possible range—to take. Third, and crucially, the degree to which the effects of such manipulations of the S-representational vehicle’s structure are actually functional should depend causally on how well those manipulations and their outcomes resemble targets. That is, if they are to successfully guide action or cognition, the internal manipulations need to actually resemble or simulate how corresponding target processes would unfold.

Take the rat’s spatial navigation system again. First, it has been suggested that place cells in a hippocampal spatial map can fire in sequences in a purely off-line manner, e.g., when the rat is asleep or is planning a route (see Johnson and Redish 2007; Pfeiffer and Foster 2013; Shea 2014; Miłkowski 2015). The map is internally manipulated and the firing sequences correspond to routes that the rat could take when navigating an actual territory. Second, these manipulations are functional for the navigational mechanism in that they (presumably) serve as a basis for route planning. Perhaps alternative routes leading to a reward are simulated in order to select one that is the shortest (Johnson and Redish 2007; Shea 2014). Third, this off-line planning is effective to the degree to which internal simulations can be actually projected to actual interactions with the environment. That is, we could manipulate the rat’s ability to effectively plan short, cost-effective routes through the environment by intervening in the degree to which its hippocampal map (and processes or manipulations performed over it) resembles structurally the terrain (and possible routes that the rat could take in it). In other words, if the rat is to be successful at planning, the unfolding of simulated actions should resemble how corresponding actions would unfold if the rat was to actually engage in them.

By concentrating on the off-line use, we do not mean to suggest that S-representations are *restricted* to the domain of off-line cognition (see also note 8). The satellite navigation example mentioned before is a case in point: here, the S-representation controls an ongoing, direct interaction with the world (if this case still counts as off-line, then only in some rather minimal sense). This point can be generalized to encompass purely subpersonal cases. Mechanisms can use S-representations to regulate on-line interactions with the environment. Imagine a cognitive system whose internal states change concurrently to changes in the external environment, and control behavior so that it is adaptive given the circumstances. Someone might, mistakenly, consider it to be a detector system, not that different from a simple thermostat. However, when we investigate the system’s workings, it turns out that its internal machinery is cut off from the target; it has no sensory apparatus. What explains its successful behavior is that it has an internal structure that continuously simulates the changing environment. This simulation is not a matter of responding to the target. Rather, it is an endogenously controlled process whose unfolding resembles the relevant dynamics in the environment, enabling the system to behave in accordance with the world it inhabits. The best, and, in fact, the only way to explain how the system manages to cope with the environment is to point to similarity between its internal processes and processes in the environment. Hence, despite working in a purely on-line manner, the system in question turns out to employ an S-representation of its environment.

Lastly, it needs to be noted that no real-life S-representational system, even one whose cognitive processes unfold in a purely on-line manner, would work if it were completely unresponsive to the changes in the external environment. It would be impossible for such an encapsulated agent to detect and correct errors in the endogenous simulation of the environment, which could lead to catastrophic consequences. It is much more reasonable to postulate a *mixed* strategy which combines detector-based and S-representation-based ways of dealing with the environment on-line (as is the case in satellite-based navigation). What we mean is a system that simulates the environment but is at the same time equipped with response-selective detectors. The internal model could make predictions about the way the detectors will be affected by states of the world, with the mismatch between that prediction and feedback that is actually generated serving as a way of “measuring” the representational error. This sort of prediction-error-based cognitive strategy is postulated by recent predictive processing approaches to cognition (Clark 2013; Friston and Stephan 2007; Friston 2010; Hohwy 2013). According to the predictive processing story, on-line perception and action are underpinned by an internal generative model which encodes the causal-probabilistic structure of the organism-external environment (Gładziejewski 2016b). The model is constantly updated in a way that aims at simulating the ongoing changes in the environment, and it constantly predicts incoming sensory stimuli (hence the qualification “generative”). Updating and prediction are endogenous in nature, as the updating crucially depends on pre-stored likelihood and prior probability distributions, and the predictions are trafficked in a top-down manner. Thus, the generative model constitutes an endogenous source of control of perception and action. However, as mentioned, the process of internal simulation could go catastrophically astray if it were impossible for it to get corrected in case of error. And here is where detectors come into play. In predictive processing, the sensory system is reliably causally dependent on the environment (hence, acts as a detector of sorts), and the difference between its actual states and internally generated predictions results in a prediction error signal, which is propagated in a bottom-up manner. This way the internal model can be corrected in light of the prediction error. In other words, the S-representation (the generative model) and detectors (the sensory apparatus) work together. To generalize this point, although S-representations are not detectors, they will sometimes need detectors to help them with their representational duties.

## Conclusions

In this paper, we attempted to clarify the claim that internal representations are S-representations. First, we proposed a mechanist-interventionist interpretation of the idea of similarity as an “exploitable” relation. This interpretation appeals to the causal role that similarity plays in enabling the successful operation of cognitive mechanisms. Second, we provided reasons for thinking that S-representations are indeed a separate type of representation, distinct from purported indicator or detector representations. On the view that we opted for, the key to this distinction lies in the fact that (1) S-representations’ workings depend on the structural similarity in a way that is not the case with detectors, and (2) they constitute an endogenous source of control that exhibits a degree of functional freedom from states of the environment. Overall, we hope that our proposals further pave the way that leads away from seeing representations as a matter of reacting to the world detector-style, and towards the idea that representing the world is a matter of actively modeling it.

Before we close the discussion, there is one last issue that merits mention. Some authors have argued that the domain in which S-representations can be explanatory is restricted to low-level cognition and that S-representations are not quite suited to explain more sophisticated, human-level cognitive capacities (Morgan 2014; see also Garzón and Rodríguez 2009). Notice, however, that our approach assumes relatively minimal, empirically uncommitted criteria of what counts as an S-representation. Because of this, our criteria can be met by internal structures that vary, perhaps drastically, in terms of their cognitive sophistication. There are a couple dimensions along which there could be such variance. First, the vehicles of S-representations can vary in their relational complexity (and there should be corresponding variance in the complexity of their representational objects). Second, the manipulations performed over those vehicles can vary in their dynamic or computational complexity. Third, S-representations can differ in how decoupled from the environment they are; i.e., they can function in a way that is more or less off-line. Fourth, perhaps a case could be made that flexibility and context-dependence of components that act as consumers of S-representations can vary (see Cao 2012). Now, if we agree that S-representations differ along those dimensions, what we end up with is a continuum of S-representations of increasing sophistication. If this is a workable position—and we see no reason to doubt this—then it should no longer be mysterious how S-representations could underlie both simple and phylogenetically old cognitive capacities, as well as complex capacities that are phylogenetically new and perhaps even human-specific, such as reasoning, imagery, or mental time travel. Roughly, more sophisticated cognitive functions are underpinned by more sophisticated S-representations. In fact, our own empirical bet is that human-level off-line cognition is largely a matter of being equipped with highly sophisticated S-representations—S-representations that actually earn the status of “mental models.”

## Footnotes

1 Although structural similarity has been variously defined, usually the definitions revolve around the common idea that the relation in question is constituted by the existence of a (at least partial) structure-preserving mapping between two entities. In the present paper, we will assume the following definition (O’Brien and Opie 2004, p. 11): Suppose SV = (V, ℜV) is a system comprising a set V of objects, and a set ℜV of relations defined on the members of V. (…) We will say that there is a second-order [structural] resemblance between two systems SV = (V, ℜV) and SO = (O, ℜO) if, for at least some objects in V and some relations in ℜV, there is a one-to-one mapping from V to O and a one-to-one mapping from ℜV to ℜO such that when a relation in ℜV holds of objects in V, the corresponding relation in ℜO holds of the corresponding objects in O.

2 As one reviewer pointed out to us, there are two ways of understanding the vehicles of S-representations. On the first interpretation, the vehicle is the whole S-representation, e.g. a model or a map as such. On the second interpretation, endorsed by Ramsey (2007), only components of larger structures (say, symbols placed *within* a map) are treated as the S-representational vehicles. These components represent by serving as stand-ins for their targets within a larger structure. Now, these two approaches are closely related, as the possibility of treating components as stands-ins presupposes the existence of structural similarity between the larger representing structure and the represented structure. We think that that the choice between the two interpretations should be a matter of one’s explanatory or theoretical agenda. In the present paper, our concern is with the role played by structural similarity. This relation of interest for us holds between the relational structure of the representation as whole and some represented target. Therefore, here, we choose to treat the whole representing structure as the vehicle of S-representation. We admit, though, that given different aims, the component-centered interpretation might be preferable.

3 Following Carl Craver’s (2009) work, we take it that the interventionist account of causal relevance can be reconciled with the neomechanist view of explanation. More specifically, we assume that the component’s contribution to the function of the mechanism as a whole can be understood in terms of this component’s being causally relevant for the function in question, where causal relevance is construed along the interventionist lines.

4 The variables and different values they take admit many ontological interpretations, i.e., they could stand-in for properties, processes, events, states of affairs, etc.

5 It needs to be stressed that the similarity exists purely in virtue of the existence of the structure-preserving mapping between the relata (see note 1). O’Brien and Opie (2004) call this a “second-order” resemblance, as it is not required that the relata, i.e., structures related by way of similarity, are of the same physical type. For example, a structure of a *spatial* arrangement and directionality of arrows on a diagram may resemble a structure of *causal* relationships in some domain. Analogously, in our hippocampal map example, the similarity does not have hold between the spatial structure of the environment and the spatial layout of neurons. Rather, the resemblance holds between a spatial structure and a neural co-activation structure which implements a coordinate space (see Shea 2014).

6 Of course, even in this case the causal relation between the two variables only holds within a certain set of background circumstances. For example, X can be said to be causally relevant to Y only if other (nonrepresentational) parts of the representation-involving mechanism are working properly. Nonetheless, we take it that the class of conditions under which *this* causal relation remains stable is wide enough for this relation to be of scientific value.

7 We are indebted to an anonymous reviewer for urging us to include this difference in our discussion.

8 Importantly, we are *not* claiming that the possibility of off-line use *itself* is what distinguishes S-representations from detectors. Rather, we treat this fact about S-representations as *resulting* from them being functionally disconnected from their targets. Our claim is simply that, from an engineering standpoint, S-representations are naturally poised to subserve off-line cognition. However, we do not want to wholesale deny that there may be *some* sense in which indicators could function off-line, e.g. when the causal chain that leads from the target appearing to the detector entering some state is so long or slow that once the detector enters this state, the target is no longer present in the environment.
